# Congenital lung malformations: an ongoing controversy

**DOI:** 10.1308/003588412X13373405387735

**Published:** 2013-03

**Authors:** RT Peters, DM Burge, SS Marven

**Affiliations:** ^1^Central Manchester University Hospitals NHS Foundation Trust, UK; ^1^University Hospital Southampton NHS Foundation Trust, UK; ^3^Sheffield Children’s NHS Foundation Trust, UK

**Keywords:** Bronchopulmonary sequestration, Cystic adenomatoid malformation of lung, congenital, Infant, Child, Survey

## Abstract

**Introduction:**

Congenital lung malformations are rare lesions that are most commonly diagnosed antenatally. Management of such lesions, particularly those that are asymptomatic, remains controversial. We undertook a survey to ascertain current practice of surgeons in the UK and Ireland.

**Methods:**

All consultant members of the British Association of Paediatric Surgeons were asked to complete a survey on congenital lung malformations with respect to antenatal management, symptomatic and asymptomatic lesions, and operative techniques.

**Results:**

Responses were received from 20 paediatric surgical centres and highlighted the ongoing variability in management of such lesions, particularly those that are asymptomatic. Twenty per cent of surgeons never resect an asymptomatic lesion and twenty-four per cent always do. The remainder intervene selectively, with size being the most commonly stated indication. Most resections are undertaken via thoracotomy although 35% of surgeons use thoracoscopy for some procedures.

**Conclusions:**

National data based on congenital anomaly registers are needed to determine the natural history of these malformations and to guide future management.

Congenital lung malformation (CLM) is a term encompassing a variety of conditions including congenital pulmonary airway malformation (CPAM), formerly known as congenital cystic adenomatoid malformation, bronchopulmonary sequestration (BPS), hybrid lesions, with features of both, and congenital alveolar overdistension (CAO), formerly known as congenital lobar emphysema. They are thought to be rare lesions with the most common, CPAM, having an estimated incidence of 1 in 8,000 to 1 in 35,000.^1,2^ However, with the increasing sensitivity of prenatal ultrasonography, the incidence of CLMs may be as high as 1 in 3,000.^3^ CLMs are often detected antenatally by routine fetal anomaly scans with further information obtained in some cases by fetal magnetic resonance imaging (MRI).

Controversy surrounds both the antenatal and postnatal management of these lesions, particularly those that are asymptomatic. Some advocate non-operative, expectant management of such lesions but others prefer to operate early as prior infection may lead to morbidity and a more difficult resection.^4,5^ In addition, there may be a malignant potential to CPAMs, giving further indication to those who advocate early resection.^6^ A survey of practice in Canada demonstrated a lack of consensus among surgeons, even in the same centre.^7^ We designed the present survey to ascertain the current practice of consultant paediatric surgeons in the UK and Ireland.

## Methods

A 50-question survey was devised and entered into online survey software (QuestionPro, Seattle, WA, US). A link to this survey was emailed to all consultant members of the British Association of Paediatric Surgeons in March 2011, inviting them to complete it. The survey concerned CLMs including CPAM, BPS and CAO. Other lesions including vascular and lymphatic anomalies and bronchopulmonary foregut malformations were not included in the questionnaire. Respondents who answered that they never dealt with children with CLMs in their practice did not complete the rest of the survey. Topics covered by the survey included background, antenatal management, asymptomatic lesions, symptomatic lesions and operative intervention.

## Results

A total of 72 surgeons undertook the survey out of a consultant membership of 140 (51% response rate). Thirty indicated that they did not manage children with CLMs. There were 8 incomplete returns, leaving 34 complete responses. Where the number of responses does not equate to the number undertaking the survey, this is because some respondents did not answer that question or were not shown that question owing to the use of branching in the survey design. An example of branching is that further questions on antenatal counselling were posed only to those who stated that they undertook such counselling.

### Background

Responses were received from 20 paediatric surgical centres in the UK and Ireland. In two of these, CLMs are managed exclusively by paediatric cardiothoracic surgeons. In the remaining centres, paediatric surgeons manage these children. In two centres, this is in collaboration with paediatric cardiothoracic surgeons, in two centres with adult cardiothoracic surgeons and in four centres with respiratory physicians. The median number of surgeons treating children with CLMs in each centre was two.

Surgeons responding to this survey had been consultants for an average of 12 years. Nineteen respondents declared a subspecialty interest in thoracic surgery. Ten had worked in an adult cardiothoracic unit at registrar level or above for an average of nine months (range: 1.5–12 months). Six had completed a fellowship or subspecialty training in paediatric thoracic surgery.

### Antenatal management

Twenty-five respondents (74%) undertake antenatal counselling for CLMs (Table 1). The majority cover issues such as infection, respiratory distress and spontaneous resolution in their counselling. Seventeen (68%) discuss the malignant potential of lesions. Antenatally, two (8%) discuss the dilemma presented by the asymptomatic child.

**Table 1:** Antenatal counselling: percentage of surgeons who discuss each issue (*n*=25)

Issue	Percentage
Infection	96%
Respiratory distress	92%
Spontaneous resolution	88%
Malignancy	68%
Vascular effects	68%
Air leak	56%
Miscarriage	48%
Neonatal mortality	8%
Dilemma of asymptomatic child	8%

Of the 20 centres, 14 reported that fetal MRI is used in some cases.

When asked specifically whether they believed that children with CPAMs have an increased risk of malignancy (no matter how small), 20 respondents believed they do and 12 that they do not.

Fetal interventions that have been used for mothers counselled by respondents (in order of frequency) included thoracoamniotic shunts, therapeutic amniocentesis, puncture of intrathoracic cysts/hydrothorax and lasering to feeding vessel(s) to sequestration.

In four centres, all deliveries occur in the tertiary hospital. Ten centres reported that selected cases only are delivered in the tertiary setting. Three centres gave responses for both routine delivery in tertiary care and selective delivery in tertiary care, perhaps reflecting differing practice between consultants in these centres.

### Asymptomatic lesions

Some form of imaging is favoured by the majority of surgeons for assessment of asymptomatic lesions, including plain chest radiography by 33 (97%) and/or computed tomography (CT) of the thorax by 31 (91%). Less than 15% consider the use of other modalities in certain cases, including MRI, chest ultrasonography, upper gastrointestinal contrast study and echocardiography.

Approximately half of surgeons who perform CT for asymptomatic lesions do so at around six weeks of life, with the remainder at a mean of six months (range: 3–12 months). Of those performing CT, 84% use intravenous contrast routinely.

For the purposes of this survey, disappearing lesions were defined as CLMs that have been detected early on antenatally but diminish subsequently in size on serial ultrasonography until they are no longer visible. Twenty-four respondents (70%) undertake postnatal imaging on children with such lesions, five (15%) do not and four (12%) stated that they had never seen such a lesion. Of the 24 surgeons undertaking imaging, 3 would always resect them if still present and 4 would never consider resecting them. Of the remaining 17, factors that would influence their decision to operate include size, parental wishes, whether the lesion was macrocystic, whether it was symptomatic and discussion in the multidisciplinary team meeting.

Seven respondents (21%) always resect asymptomatic lesions and eight (24%) never resect them. The remaining 19 surgeons (56%) reported they sometimes do. Sixteen indicated that the size of the lesion would influence this decision. Very few gave a particular size above which they would resect a lesion as they stated that other factors in combination would influence their decision to offer resection. For all types of CLM, the most consistently reported factors influencing the decision to resect an asymptomatic lesion are size, parental anxiety and the desire for a tissue diagnosis (and in particular for CPAMs) if the lesion is macrocystic.

None of those removing asymptomatic lesions do so before the child is three months old. Nineteen respondents (56%) resect before the age of one year with the remainder resecting later than this.

The follow-up policy for asymptomatic lesions that have not been resected varies widely. Ten respondents either leave follow-up to the respiratory physicians or ask the patient to re-present to the general practitioner should symptoms develop. The remainder maintain regular follow-up for a variable time (1–10 years) with inconsistency of when imaging (if at all) is repeated. Many advise repeat chest radiography or CT at six months or one year of age. One respondent refers children with sequestrations not resected to the cardiology team for follow-up.

### Symptomatic lesions

All respondents except one recommend resection of symptomatic CPAM, BPS, hybrid and CAO lesions. If symptoms are those of infection, 11 respondents (42%) resect after 1 infection and 8 (31%) after 2 infections, with others giving ranges typically of 1–2 or 2–3 infections. Other comments included whether the infection was localised to the area of the lesion.

Eighteen respondents (56%) have treated at least one child with a primary lung tumour. Twelve of these reported pleuropulmonary blastoma (PPB). Other tumours that had been seen included bronchogenic carcinoid, sarcoma, myofibroblastic tumour and anaplastic large cell lymphoma. Four of the respondents recalled a link to a CLM with these tumours. All four were PPB. Three were diagnosed on imaging as CPAM (one of these presented initially with a pneumothorax) but on resection they were histologically PPB. The final case was a five-year-old girl with symptoms since infancy. The presumed diagnosis on imaging was again CPAM. On resection, the histology revealed low grade PPB with background change in that lung of CPAM/CAO. We do not have further details regarding these cases and, in particular, whether they were diagnosed antenatally.

### Operative management

Twenty-six respondents operate on CLMs. Half stated they never use peri-operative strategies to isolate the lung. The remainder commonly use a double lumen endotracheal tube, mainstem bronchial intubation and a bronchial blocker.

Access to the thoracic cavity is most commonly by a thoracotomy, sparing the serratus anterior but cutting the latissimus dorsi. Twelve respondents (46%) use a full muscle sparing thoracotomy at least some of the time. Thoracoscopy is used by nine respondents (35%) ‘sometimes’ or more often. Surgeons using thoracoscopy most commonly use vessel-sealing devices or endostapling devices for dividing pulmonary vessels. For the lobar bronchus, either endostapling devices or sharp division with intracorporeal suturing are most commonly used.

Following resection, 20 respondents (77%) always leave a chest drain in situ, with the rest often or rarely. Of those who often leave a drain, their decision is based on the presence of an air leak at the end of the procedure, the extent of fissure development and whether the patient will be ventilated post-operatively.

## Discussion

Practice varies widely regarding the management of CLMs in the UK and Ireland. The rarity of these conditions and the often polarised views of those treating such children mean that high level evidence to inform optimum management will be difficult to obtain. In particular, management of asymptomatic lesions differs between surgeons and, with regard to CPAMs, this appears to be in part driven by beliefs regarding malignant potential.

As antenatal diagnosis of such lesions by obstetricians continues to become more sensitive, there is a need for more data to determine the natural history of these conditions. This is likely to lead to a growing cohort with lung lesions detected antenatally that would have remained asymptomatic and undetected previously.^3^ Series from individual centres contribute^2,8^ but a registry with much larger numbers may help to address some of the unanswered questions. In the UK, regional congenital anomaly registers have reported cohorts but, currently, no complete national data exist.^9,10^ Clearly, national outcome data would help to inform the debate, and would possibly settle some of the controversies and narrow the wide variation in practice. This would benefit parents and inform those counselling them as well as benefitting surgeons contemplating intervention and, of course, the patients throughout their lifetime.

A UK registry of antenatally diagnosed CLMs ought to be possible given that so few centres and surgeons deal with these congenital anomalies. Such a register would need participation from both paediatric and cardiothoracic surgeons as well as respiratory physicians and obstetricians, for follow-up of surgically and non-operatively managed children.

In the majority of centres, an average of two surgeons deal with these conditions (in four centres this is in collaboration with respiratory physicians). In some centres, their management is exclusively by paediatric cardiothoracic surgeons. Currently, there are ten paediatric cardiothoracic surgical centres in England and it is likely that these will be reduced to either six or seven centres.^11^ This may mean that there will be a greater need for paediatric surgeons to take on the management of CLM and cardiothoracic support may decrease.

Training of paediatric surgeons in thoracic surgery is incorporated in the current six-year training programme. Some individuals choose to focus on this in latter training years and, in addition, some undertake fellowships after completion of training or out-of-programme attachments in adult or paediatric thoracic centres. Completion of such specialised training will not necessarily lead to a consultant post in this subspecialty. A more formalised approach to training in paediatric thoracic surgery, possibly in conjunction with adult or paediatric cardiothoracic units, should be considered by the specialty advisory committees on training.

## Conclusions

The management of CLMs deserves more attention but until we have good quality data on the natural history of these conditions, practice will continue to be driven by individual surgeons’ personal preference or expertise.
